# Extraction of Phenolic Compounds with Antioxidant Activity from Strawberries: Modelling with Artificial Neural Networks (ANNs)

**DOI:** 10.3390/foods10092228

**Published:** 2021-09-20

**Authors:** Iman Golpour, Ana Cristina Ferrão, Fernando Gonçalves, Paula M. R. Correia, Ana M. Blanco-Marigorta, Raquel P. F. Guiné

**Affiliations:** 1 Department of Mechanical Engineering of Biosystems, Urmia University, Urmia P.O. Box 5756151818, Iran; imangolpour@gmail.com; 2 CERNAS Research Centre, Department of Food Industry, Polytechnic Institute of Viseu, 3504-510 Viseu, Portugal; aferrao@esav.ipv.pt (A.C.F.); fgoncalves@esav.ipv.pt (F.G.); paulacorreia@esav.ipv.pt (P.M.R.C.); 3 Department of Process Engineering, Universidad de las Palmas de Gran Canaria, 35017 Las Palmas de Gran Canaria, Spain; anamaria.blanco@ulpgc.es

**Keywords:** strawberry, total phenolic compounds, antioxidant activity, artificial neural networks (ANNs)

## Abstract

This research study focuses on the evaluation of the total phenolic compounds (TPC) and antioxidant activity (AOA) of strawberries according to different experimental extraction conditions by applying the Artificial Neural Networks (ANNs) technique. The experimental data were applied to train ANNs using feed- and cascade-forward backpropagation models with Levenberg-Marquardt (LM) and Bayesian Regulation (BR) algorithms. Three independent variables (solvent concentration, volume/mass ratio and extraction time) were used as ANN inputs, whereas the three variables of total phenolic compounds, DPPH and ABTS antioxidant activities were considered as ANN outputs. The results demonstrate that the best cascade- and feed-forward backpropagation topologies of ANNs for the prediction of total phenolic compounds and DPPH and ABTS antioxidant activity factors were the 3-9-1, 3-4-4-1 and 3-13-10-1 structures, with the training algorithms of trainlm, trainbr, trainlm and threshold functions of tansig-purelin, tansig-tansig-tansig and purelin-tansig-tansig, respectively. The best R^2^ values for the predication of total phenolic compounds and DPPH and ABTS antioxidant activity factors were 0.9806 (MSE = 0.0047), 0.9651 (MSE = 0.0035) and 0.9756 (MSE = 0.00286), respectively. According to the comparison of ANNs, the results showed that the cascade-forward backpropagation network showed better performance than the feed-forward backpropagation network for predicting the TPC, and the FFBP network, in predicting the DPPH and ABTS antioxidant activity factors, had more precision than the cascade-forward backpropagation network. The ANN technique is a potential method for estimating targeted total phenolic compounds and the antioxidant activity of strawberries.

## 1. Introduction

Strawberries (*Fragaria ananassa*), a member of the Rosaceae family, are one of the significant sources of phenolic compounds, along with antioxidant and antiproliferative activities of fruits. They are widely consumed due to their nutritional content and flavour [1,2]. It has been reported that the antioxidant properties of strawberries are due to their high content of total phenolic compounds rather than vitamin C [2]. The total phenolic compounds available in strawberries have an impact on their quality, contributing to organoleptic and sensorial properties and also to health properties [3]. Strawberries, because of these different health advantages in addition to their nutritional value, have seen increasing worldwide production and consumption and are thus known as the first most significant soft fruit species [4]. Useful polyphenols such as hydrolysable (ellagitannins and gallotannins), flavonols, anthocyanins and condensed tannins are present in strawberry fruits [5,6].

Strawberries, due to their high antioxidant levels and the beneficial impacts on human health, prevent several chronic pathologies, like cancer, obesity, cardiovascular diseases, inflammation-related pathologies and Alzheimer’s disease [7,8,9]. Research works showed that phenolic compounds have been replacing synthetic antioxidants and antimicrobial agents in food crops because of their prominent antimicrobial activities, which could be utilized in the process of functional food formulations as well as pharmaceuticals for health-promoting impacts [10]. 

In food industries, the extraction process is one of the crucial steps in recovering phenolic compounds [11]. It should be noted that this process can be carried out using several methods to extract the phenolic compounds. In addition, ideal extraction techniques depend on the kind of food product under analysis [12]. Some of the techniques that can be applied to extract phenolics include infusion, percolation, digestion, decoction, maceration, Soxhlet extraction, aqueous alcoholic extraction by phytonics processes, ultrasound extraction, fermentation, supercritical fluid extraction and countercurrent extraction [3]. Among these techniques, solid–liquid extraction has been extensively utilized to separate various compounds during the recovery of antioxidant phenolic compounds; the efficiency of the extraction process can be affected by parameters like the solvent/solid ratio, the extraction time, the type and the concentration of the solvent, and temperature [13]. Naczk and Shahidi [14] demonstrated that extraction times longer that 24 h could increase the oxidation process of phenolic compounds.

One of the important opportunities for researchers, faced with time-consuming and costly methodologies, to acquire reputable information for various operating conditions involves new products and processes obtained through developing the science of soft computing. Artificial intelligence systems (AISs) like artificial neural networks (ANNs) could be a powerful tool to predict nonlinear system data for overcoming these concerns [15]. ANN as an intelligent alternative approach for solving engineering problems has been used to adjust multi-variable nonlinear functions [16,17]. The ANN modelling method has been used to predict food properties and model many processes in food industries, such as the antioxidant activity of bananas [18], tea [19], essential oils [20] and beetroot [21]. In all these cases, the antioxidant properties have been modelled with good accuracy through the application of ANN modelling. On the other hand, Gutés et al. [22] used ANN analysis for determining different phenols using an electronic tongue, which combines biosensor measurements with chemometric tools.

Estimating the value of extractable total phenolic compounds (TPC) and the antioxidant activity (AOA) of strawberries is extremely advantageous, not only for the consumption of fruits and their derivatives but also for possible industrial uses. In this case it necessarily requires knowledge of the related phenolic profiles and traits of the samples, which will help to determine the most appropriate sample for industrial scale extraction aimed at increasing the amount of compounds with antioxidant activity. Although ANN modelling has been applied in the extraction prediction of fruits, to the authors’ knowledge, there has been no p previous evaluation of the total phenolic compounds and antioxidant activity of strawberries using artificial neural networks (ANNs). Thus, the aim of this research is to model the effect of different experimental extraction conditions, such as time, volume/mass ratio and extracting solution, using artificial neural networks (ANNs) on the TPC and AOA, using two methodologies to evaluate the latter (ABTS and DPPH). Our work intends to optimize the extraction method based on the above-mentioned input variables, using the strawberry as our sample matrix. Our aim is to predict maximum TPC and AOA under the best operating conditions, for example, minimum use of ethanol and increased use of water.

## 2. Materials and Methods

### 2.1. Sample Preparation and Extraction Methodology

The strawberry samples used in the present work were acquired at a local market and transported to the laboratory, where they were peeled and ground for obtaining a uniform mass. A 5 g sample was taken from the ground strawberry mass and then used to extract the phenolic compounds. The extraction procedure consisted of several assays, and for each three extraction steps were performed successively on the same sample. For each assay, different conditions were used, namely different extraction times (from 20 to 60 min), different solvent concentrations (aqueous solutions of methanol, varying from 40 to 100%) and different solvent volume to sample mass ratios (varying from 6 to 12 mL/g).

For experimental planning of the assays, a 2**(3) central composite design (nc = 8, ns = 6, nc0 = 2, ns0 = 1) was used, and consequently 18 experimental runs were performed.

The extracts obtained were used to quantify the phenolic composition and the antioxidant activity.

### 2.2. Chemical Analyses

The content of TPC in the extracts was determined by the Folin-Ciocalteu reagent, by adaptation of the method by Gonçalves et al. [23] and Guiné et al. [24]. A total of 0.125 mL of each sample was added to 0.75 mL of deionized water and 0.125 mL of the Folin-Ciocalteu reagent. Then, the solution was left to stand for 6 min; after this, 2 mL of a 5% (m/v) solution of sodium carbonate was added, and the mixture was left to rest again for 90 min at room temperature in the dark. A calibration curve was obtained with standard solutions of gallic acid, and the absorbance was measured in a spectrophotometer at 760 nm. The results were expressed as milligrams of gallic acid equivalent (GAE) per gram of fresh sample, being a mean of three measurements.

The AOA was determined using two assays: the free radical 2,2′-azino-bis(3-ethylbenzthiazoline-6-sulphonic acid (ABTS)) and the free radical 2,2-Diphenyl-1-picrylhydrazyl (DPPH). The results were based on the percentage of inhibition, compared to a standard antioxidant (Trolox) in a dose–response curve, being expressed in µmol of Trolox equivalent (TE) per gram of fresh sample. 

The ABTS method is based on the abilities of different substances to scavenge the ABTS^+^ radical compared with a standard antioxidant (Trolox: 6-hydroxy-2,5,7,8-tetramethylchroman-2-carboxylic acid). For the assay, ABTS^+^ radical was prepared by mixing an ABTS^+^ stock solution (7 mM in water) with 2.45 mM potassium persulfate. This mixture was allowed to stand for 12–16 h at room temperature in the dark until it reached a stable oxidative state. The ABTS^+^ solution (1 mL) was diluted in 80 mL of ethanol or buffer solution prior to utilization. In a tube was placed 2 mL of ABTS^+^ solution with 0.1 mL of sample, and after agitation it was left to rest in the dark for 15 min [24,25]. Then, the absorbance was measured at 734 nm to assess the percentage of inhibition, using a calibration curve previously obtained. 

In the DPPH method, 100 µL of sample and 2 mL of DPPH previously prepared with methanol were added to a tube, which was placed in the dark at room temperature for 30 min. After that, the absorbance was measured in a spectrophotometer at 515 nm. The results were calculated from the percentage of inhibition of each sample as compared to Trolox as the standard antioxidant in a dose–response curve [24,26,27]. 

The analyses for antioxidant activity were performed in triplicate for each of the extracts analysed.

### 2.3. ANN Based Modelling

**Figure 1:**
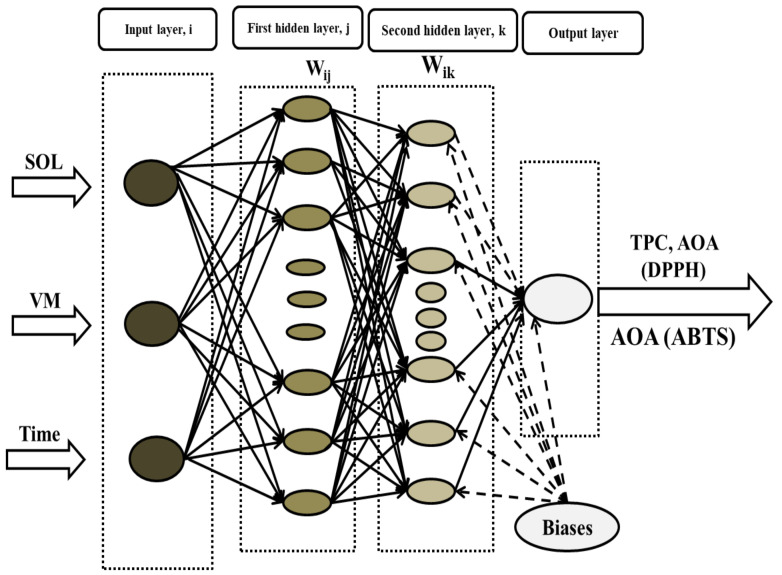
Proposed architecture of MLP ANN.

A multilayer perceptron (MLP), with two models of feed-forward backpropagation (FFBP) and cascade-forward backpropagation (CFBP) in the ANN model generated by the toolbox of Neural Network (NN) used in MATLAB software R2018b were created and tested with one and two hidden layers under architectures of 3–x–1 and 3–x–y–1 and different neurons to estimate the outputs. The input and output neurons of the networks with developed topology with two hidden layers are illustrated in Figure 1. The input parameters of the ANNs consisted of the levels of time, volume/mass ratio and solvent, while the output variables for prediction were the values of the TPC, AOA (DPPH) and AO (ABTS) of strawberries. Table 1 shows the boundaries and levels for the three inputs and three outputs applied.

Moreover, several topologies were evaluated by application of the raising method for changing the available neurons of ANNs. The training process of ANNs was done based on Levenberg-Marquardt (LM) (trainlm code) and Bayesian regulation (BR) (trainbr code) algorithms for updating network weights. The evaluation process of ANNs for each output parameter was done individually for facilitating the training process of the neural networks (NNs) and analysis of the obtained results. The transfer functions used to obtain the best network structure were linear function (PUR), logarithmic sigmoid (LOG) and hyperbolic tangent sigmoid (TAN), according to the following equations [15]:

(1) $$Y_{j}=X_{i}\;\;\;\;\;(\mathrm{purelin})$$

(2) $$Y_{j}=\frac{2}{(1+\exp(-2X_{j}))-1}\;\;\;\;\;(\mathrm{Tansig})$$

(3) $$Y_{j}=\frac{1}{1+\exp-X_{j}}\;\;\;\;\;(\mathrm{Logsig})$$

where *Xj* is computed as follows:

(4) $$X_{j}=\sum_{i=1}^{m}W_{ij}\times Y_{i}+b_{j}$$

where m is the number of neurons in output layer, *W_ij_* is the corresponding weight between *i^th^* and *j^th^* layers, *Y_i_* is the *i^th^* output neuron, *X_j_* is the *j^th^* input neuron and *b_j_* is the bias of the *j^th^* neuron for the related networks.

With the goal to estimate the antioxidant activity of foods based on phenolic contents using the ANN technique, the ANN-based model was created. The total set of sample data was divided into two subsets to train ANN and test the estimation capability. In order to train the subset, 70% of samples were randomly selected, while the testing subset had 30% of the samples. The details of the ANN model are shown in Table 2.

### 2.4. Data Normalization and Error Evaluation

For improving the capability and performance of the ANN model in recognizing relations among related inputs and outputs, guaranteeing the convergence and process stability, data normalization was done in the first step in the ANN modelling to forecast the outputs with respect to the following equation [15,28]:

(5) $$X_{norm}=\frac{X_{r}-X_{\min}}{X_{\max}-X_{\min}}$$

where *X_r_* and *X_norm_*, represent the values of measured and normalized data, respectively, and *X_min_* and *X_max_* are the minimum and maximum values of the measured factors, respectively.

The best network performance was statistically gained by the mean square error (MSE) and the determination coefficient (R^2^), which were obtained using following formulas [3,29,30]:

(6) $$MSE=\frac{1}{n}\sum_{k=1}^{n}{\left(S_{k}-T_{k}\right)}^{2}$$

(7) $$R^{2}=1-\frac{\sum_{k=1}^{n}\left[S_{k}-T_{k}\right]}{\sum_{k=1}^{n}\left[S_{k}-\frac{\sum_{k=1}^{n}S_{k}}{n}\right]}$$

where *S_k_* is the predicted output values of the network for the *k^th^* dataset, *T_k_* is the target output for the *k^th^* dataset and n is the number of specific training patterns.

## 3. Results and Discussion

### 3.1. Experimental Results

Table 3 presents the results obtained for the studied properties, total phenolic compounds and antioxidant activity (DPPH and ABTS methods), considering variable experimental conditions: time varying from 40 to 60 min, volume of extracting solution to mass ratio (V/M) varying from 9 to 12 mL/g and concentration of the solvent varying from 40% water (60% ethanol) to 100% water (0% ethanol). Although more combinations were performed for the ANN modelling, 18 combinations to be precise, the seven presented in Table 3 are the most representative for a general overview of the problem at a macroscopic scale, which allow a better visualization of the effect of the different conditions on the measured properties of the extracts. The run for central point conditions (40 min extraction time, 9 mL/L volume/mass ratio and 70:30% water: ethanol in extracting solution) was repeated several times according to the experimental design technique. For each of the runs, a total of three measurements were made for each property, and the values presented result from the calculation of the average and standard deviation of those measurements. The results in Table 3 indicate that the highest TPC concentration (1.494 mg GAE/g) was obtained for extraction with a solution of 70% water to 30% ethanol, for a V/M ratio of 12 mL/g and a 40 min extraction time. However, the value obtained for the same conditions but extracting with 100% water was very similar (1.457 mg GAE/g). Bearing this in mind, it would be preferable to choose the latter option of not using any organic solvents, i.e., perform the extraction only with water. When looking at the antioxidant activity, the results obtained with the two methods were quite different, which is derived from the chemical nature of the substances and the reactions involved. While for the experiments made with the DPPH methods the results are very similar for all tested conditions, the results for ABTS are quite dependent on the variability of the processing parameters. In this way the highest value of AOA for the DPPH method (1.297 mg TE/g) was obtained for the 40 min extraction time, with V/M equal to 9 mL/g and a 70% concentration of solvent; again, the difference when using 100% water was minimal (1.271 mg TE/g), thus showing a very similar trend to that of the TPC. With regard to the ABTS AOA, the highest value (3.368 mg TE/g) was obtained for only the 20 min extraction, with a V/M ratio of 9 mL/g and a solution with 70% water and 30% ethanol. In this case, the possibility of using 100% water as the extracting solution was not viable (1.686 mg TE/g of ABTS AOA) (Table 3). Conventional extraction of bioactive substances such as phenolic compounds or other compounds with antioxidant activity is frequently performed using organic solvents like ethanol, methanol or acetone. However, the extractions are usually executed in a batch process, requiring several steps aimed at separating the extracted components from the solvents used in order to recover the solvents. Still, this process will eventually result in extracts with residual amounts of solvent, which could sometimes limit their applicability. Moreover, these solvents can be responsible for high quantities of waste, which in most cases are potentially harmful to the environment. Therefore, for industrial applications, the use of clean solvents such as water is highly beneficial on one hand because it is cheaper and more accessible and on the other hand because it is cleaner and more environmentally friendly [31,32].

### 3.2. ANN Modelling for Prediction of TPC

**Figure 2:**
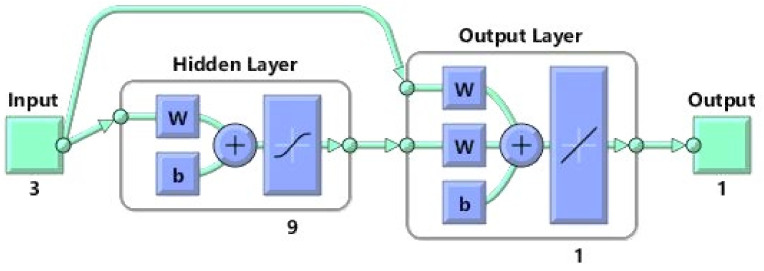
Best network topologies with the Levenberg-Marquardt training algorithm for prediction of total phenolic compounds (TPC).

The development of a neural network (NN) to predict the TPC was first done with a small network architecture, including one hidden layer, and demonstrated good results. For avoiding overfitting in ANNs, the number of related neurons for the hidden layer is raised during each session of the training process to obtain the best performance [33]. According to the obtained results, the best network structure was selected as a one-layer cascade-forward (CF) neural network type, with a topology of 3-9-1 (Figure 2). The performance of the chosen models is illustrated in Table 4, with different hidden layers and neurons. The results show that the determination coefficients (R^2^) are greater than 0.95 and the Mean Squared Errors (MSEs) are very low for the prediction of TPC. Accordingly, the models are generally very trustworthy for the dataset. As shown in Table 4, the CFBP network with a topology of 3-9-1 is the most suitable, with a threshold function of Tansig relevant to the hidden layer and Purelin for output layer, with determination coefficient and mean square error values of R^2^ = 0.9806 and MSE = 0.00470, respectively. The results demonstrate that using the threshold function of Purelin in the output layer and the Tansig function in the hidden layer had better performance, reducing the ANN error function in the prediction of TPC. 

**Figure 3:**
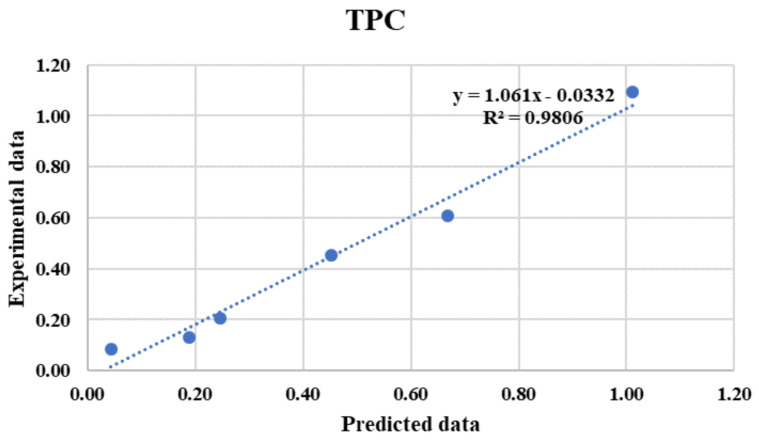
Predicted TPC values of strawberries using artificial neural networks (ANNs) versus experimental values for testing dataset.

Overall, a high correlation was found between the estimated results and targets; the mean accuracy of R^2^ = 0.9806 demonstrates that the developed network is practicable and efficient for prediction of the TPC (Figure 3). Figure 3 shows the estimated values of TPC, with the desired output values by application of the optimal ANN and the experimental values, and shows that the data points are placed around a 45° straight line, indicating the suitability of the selected multilayer feed-forward ANNs for the prediction of TPC. Accordingly, it can be seen that the TPC predicted using the optimal topology of the ANN were very close to those of experimental data. The quality and pre-processing of the training data, magnitude, type and structure of the ANN and the learning algorithm for that specific case can help to solve important problems through the application of ANN modelling [34]. Accordingly, the results showed that the backpropagation algorithm applied in this research achieved the best fit to the training data due to its available capacity of indicating non-linear functional relationships among considered inputs and targets [35]. It should be also noted that using a high number of hidden neurons for the best structure (3-9-1) obtained to predict TPC with the related threshold functions may cause overlearning of the ANN [35]. According to the high determination accuracy of the predicted dataset in the network processes, it can be concluded that the considered neural networks are capable of predicting the TPC of the strawberries. It should be mentioned that Guiné et al. [18], who studied the prediction of the phenolic contents and antioxidant activity of bananas according to four input parameters (variety, dryness state, type and order of extract) found determination coefficients between antioxidant activity and phenolic contents from 0.5833 to 0.6819, which were lower than the determination coefficients obtained for this research study.

### 3.3. ANN Modelling for Prediction of AOA (DPPH)

Table 5 shows the performance parameters of the ANN models with suitable structures and threshold functions for predicting the AOA (DPPH). The determination coefficients between the experimental and predicted outputs are generally higher than 0.95, without any sign of overfitting during the ANN training for the all obtained structures (Table 5).

**Figure 4:**
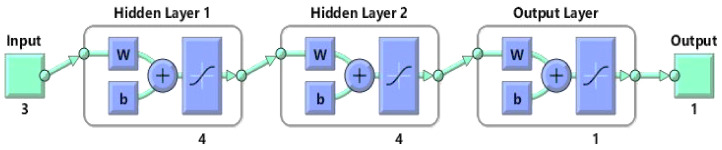
Best network topologies with the Bayesian regulation training algorithm for prediction of antioxidant activity (DPPH).

Figure 4 shows that FFBP with two hidden layers was the best ANN for prediction of AOA (DPPH). Moreover, based on the reported accuracies in Table 5, it can be concluded that the use of the Tansig threshold function used in the output layer provides the best rational choice to model non-linearities over all experiments in the prediction of the AOA (DPPH). Moreover, Purelin had good performance as a threshold function in the output layer of other ANN structures in predicting the AOA (DPPH). However, the best neural network models create the best correlations between predicted values by the ANN and the experimental values obtained in the laboratory. Therefore, there is an acceptable confidence in the analysis, considering the performance of the related models of ANNs. Overall, the best results obtained for predicting the AOA (DPPH) belonged to the FFBP network and 3-4-4-1topology, 25 epochs, and the Tansig-Tansig-Tansig threshold function with the LM training algorithm as the primary strategy. This structure resulted in MSE = 0.00350 and R^2^ = 0.9756, which shows that the selected ANN had good performance in predicting the AOA (DPPH).

With respect to the obtained results, the Bayesian regularization backpropagation algorithm (BR) utilized in the training sessions offers elimination or reduction of the exhaustive cross-validation and is more powerful than Levenberg-Marquardt (LM) as a regular backpropagation algorithm [36]. Overall, the Bayesian performance is also better than the early stopping method in the effort to obtain network generalization, especially for a small dataset [37]. The results illustrate that the ANN modelling can be applied effectively to predict AOA (DPPH), based on the considered input dataset and identified structures.

**Figure 5:**
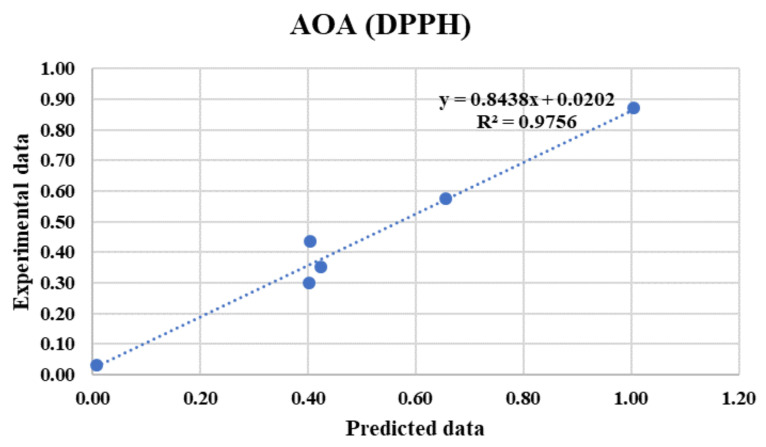
Predicted AOA (DPPH) values of strawberries using artificial neural networks versus experimental values for testing dataset.

Hosu et al. [38] predicted the antioxidant activity of Romanian red wines using data on total phenolics, flavonoids, anthocyanins and tannins and found related relative errors between the predicted and actual data of the antioxidant activities of the wines of less than 3%. The predicted values as compared to the real experimental values for AOA (DPPH) are shown in Figure 5, which confirms that the developed FFBP network is efficient and feasible and has a good performance, with suitable testing accuracy (0.9756) for prediction of AOA (DPPH). It can be shown that the predicted values of AOA (DPPH), determined using the optimal topology of ANNs, are close to those of the empirical data.

### 3.4. ANN Modelling for Prediction of AOA (ABTS)

To create the ANN-based model, the data were divided into two subsets: training and testing. The estimated and experimental datasets for the training samples were compared, and the results obtained to test the performance of the developed ANN models are presented in Table 6. Furthermore, the impact of the hidden layer number and neuron number for each hidden layer on the accuracy of the prediction can be seen from the data in Table 6.

**Figure 6:**
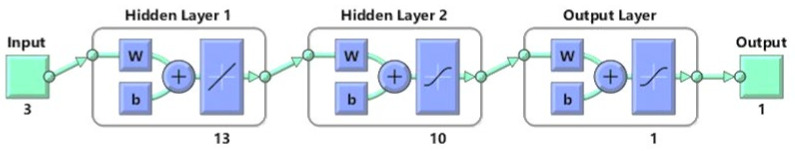
Best network topologies with Levenberg-Marquardt training algorithm for prediction of antioxidant activity (ABTS).

As shown in Figure 6, the best ANN topology and parameters were selected as 3-13-10-1 for predicting the AOA (ABTS). Table 6 illustrates the high capability of the ANNs to produce outputs similar to the experimental data. The determination coefficient (R^2^) values obtained were greater than 0.94 for the test dataset, whereas the values of MSE were very low. The results obtained indicate that the developed network could be utilized for subsequent analysis due to the acceptable performance. The results demonstrated good correlation between the predicted and experimental values for the network subsets; the best determination coefficient for prediction of AOA (ABTS) was found to be R^2^ = 0.9651 for the FFBP network, with a topology of 3–13-10-1, MSE = 0.00286, Purelin-Tansig-Tansig, and an LM training algorithm at 32 training epochs. 

**Figure 7:**
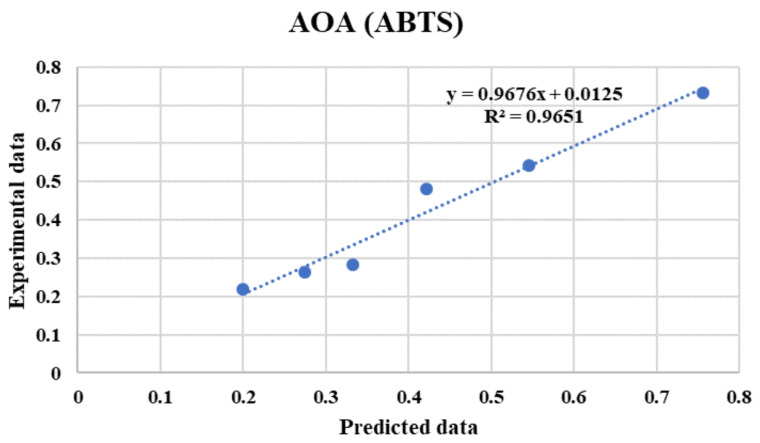
Predicted AOA (ABTS) values of strawberries using artificial neural networks versus experimental values for the testing dataset.

Figure 7 shows the relation between the predicted values by ANNs and the experimental values for the AOA (ABTS). With respect to the obtained results for this study, the maximum value of R^2^ was 0.9651 for the prediction of AOA (ABTS). Thus, neural networks (NNs) are potent tools for AOA (ABTS) modelling in different conditions, being extremely accurate and taking little time to obtain results. Cimpoiui et al. [19] used ANN modelling to predict the antioxidant activity of tea samples, with a relative error less than 0.5% based on methyl-xanthine, catechin and flavonoid content, revealing the good predictive ability of ANNs. The antioxidant activity and content of total phenolic compounds obtained in this work differed from that study; however, acceptable results were obtained, making this research successful in the case of strawberries.

This research study illustrated that ANN modelling can be applied to predict the total phenolic compounds and antioxidant activities of samples, with good determination coefficients.

## 4. Conclusions

This research study used ANN modelling techniques to estimate the antioxidant activity and total phenolic contents of strawberry samples. The feed- and cascade-forward ANN-based models were designed and trained by application of the backpropagation algorithm. The results showed that the TPC, AOA (DPPH) and AOA (ABTS) of strawberries could be predicted with a satisfactory accuracy of more than 0.94 for the training and testing subsets of data, which is the acceptable value for the developed system to be applicable in practice. Moreover, the training algorithm of Levenberg-Marquardt showed better performance than Bayesian regulation in predicting the TPC and AOA (ABTS). It should be mentioned that the CFBP model was able to predict TPC with an accuracy of 0.98, which was the highest value among determination coefficients for all developed ANNs. Overall, the findings of this research work demonstrate that the developed ANN models are promising and powerful tools that can be used instead of the mathematical models for the prediction of TPC and AOA.

In terms of practical application, these models are highly relevant, because the extraction of valuable bioactive compounds with antioxidant activity from biological matrices requires expensive and time-consuming techniques and can involve the use of organic solvents with a high environmental impact. In this way, these models can be used to predict both the amount of phenolic compounds extractable from biological samples as well as their antioxidant activity, as a function of the extraction conditions like extraction time, ratio of volume of solution/mass of sample and concentration of the extracting solution. This allows optimization of the process by maximizing the extraction of phenolic compounds and also maximizing antioxidant activity, while minimizing the use of ethanol. In this way, it is possible to choose optimal extractions without performing the actual set of time- and resource-consuming experiments in the laboratory.

Finally, it is worth noting that the applications of the extracted phenolic compounds are aimed at incorporation into food products to enhance their health-promoting properties, such as antioxidant activity, and therefore it is desirable to minimize the use of ethanol for applications in the health foods sector.

## Figures and Tables

**Table 1:** Characteristics of identified variables in input and output layers used in the model of ANNs.

Input Variables to the ANNs (Units)	Range	Output Variables for the ANNs (Units)	Range
Time (min)	20–60	Total phenolic compounds (TPC) (mg GAE/g)	1.066–1.550
Volume/Mass ratio (mL/g)	6–12	Antioxidant activity (AOA-DPPH) (mg TE/g)	0.904–1.656
Solvent (%)	40–100	Antioxidant activity (AOA-ABTS) (mg TE/g)	1.446–4.352

**Table 2:** Details of the ANN models.

No	Particulars	Specifications
1	Network type	Feed-Forward Backpropagation (FFBP) Cascade-Forward Backpropagation (CFBP)
2	Training function or Training algorithm	Levenberg-Marquardt (LM) backpropagation (TRAINLM) Bayesian regulation (BR) backpropagation (TRAINBR)
3	Adaption learning function	Gradient Descent with Momentum Weight and Bias (LEARNGDM)
4	Performance function	Mean Square Error (MSE)
5	Transfer functions	Hyperbolic Tangent Sigmoid (TANSIG) Logarithmic sigmoid (LOGSIG) Linear (PURELIN)
6	Data division	Random (Dividerand)
7	Number of input layer units	3
8	Number of output layer units	1
9	Number of hidden layers	1 and 2
10	Number of hidden layer neurons	Iterative
11	Number of epochs (Learning cycle)	1000 iterations for Levenberg-Marquardt (LM); 2000 iterations for Bayesian regulation (BR)

**Table 3:** Experimental results for TPC and AOA (DPPH and ABTS) considering different extracting conditions (values expressed as mean ± standard deviation).

Time (min)	Volume/Mass Ratio (mL/g)	Conc. Solvent (%)	TPC (mg GAE/g)	AOA-DPPH (mg TE/g)	AOA-ABTS (mg TE/g)
40.0	9.0	70.0	1.318 ± 0.105	1.182 ± 0.161	2.858 ± 0.719
20.0	9.0	70.0	1.240 ± 0.029	1.188 ± 0.070	3.368 ± 0.042
60.0	9.0	70.0	1.293 ± 0.039	1.251 ± 0.009	2.070 ± 0.041
40.0	6.0	70.0	1.457 ± 0.036	1.297 ± 0.071	2.401 ± 0.004
40.0	12.0	70.0	1.494 ± 0.072	1.201 ± 0.072	3.036 ± 0.129
40.0	9.0	40.0	1.146 ± 0.068	1.187 ± 0.084	2.277 ± 0.174
40.0	9.0	100.0	1.446 ± 0.021	1.271 ± 0.029	1.686 ± 0.156

**Table 4:** Best topologies included in training algorithms, various layers and neurons for estimation of total phenolic compounds (TPC).

Network	Training Algorithm	Threshold Function	Topology	Epoch	R^2^	MSE
**CFBP**	**LM**	**Tansig-Purelin**	**3-9-1**	**11**	**0.9806**	**0.00470**
		Purelin-Tansig-Purelin	3-6-3-1	15	0.9709	0.00620
		Tansig-Tansig	3-11-1	10	0.9783	0.00900
	BR	Purelin-Purelin-Tansig	3-5-5-1	5	0.9691	0.02490
		Purelin-Purelin	3-10-1	22	0.9599	0.03170
FFBP	LM	Logsig-Logsig-Tansig	3-20-5-1	35	0.9755	0.00510
		Tansig-Tansig-Tansig	3-10-8-1	44	0.9730	0.00980
	BR	Purelin-Purelin	3-15-1	12	0.9655	0.02780

**Table 5:** Best topologies included in training algorithms, various layers and neurons for estimation of AOA (DPPH).

Network	Training Algorithm	Threshold Function	Topology	Epoch	R^2^	MSE
FFBP	LM	Purelin-Purelin	3-7-1	9	0.9601	0.00910
		Purelin-Tansig-Purelin	3-15-9-1	15	0.9595	0.09500
	**BR**	**Tansig-Tansig-Tansig**	**3-4-4-1**	**25**	**0.9756**	**0.00350**
		Purelin-Tansig-Tansig	3-10-10-1	44	0.9734	0.00440
		Logsig-Tansig-Purelin	3-10-8-1	13	0.9695	0.00610
		Tansig-Purelin	3-3-1	4	0.9633	0.00830
CFBP	LM	Tansig-Tansig-Tansig	3-12-6-1	18	0.9520	0.09900
	BR	Purelin-Purelin-Purelin	3-7-3-1	27	0.9525	0.09400

**Table 6:** Best topologies included in training algorithms, various layers and neurons for estimation of AOA (ABTS).

Network	Training Algorithm	Threshold Function	Topology	Epoch	R^2^	MSE
**FFBP**	**LM**	**Purelin-Tansig-Tansing**	**3-13-10-1**	**32**	**0.9651**	**0.00286**
		Tansig-Tansig	3-8-1	18	0.9622	0.00260
		Tansig-Purelin -Purelin	3-5-3-1	9	0.9512	0.00415
	BR	Logsig-Purelin-Tansig	3-8-8-1	22	0.9601	0.00370
		Tansig-Purelin-Tansig	3-3-3-1	14	0.9555	0.00500
		Purelin-Tansig	3-6-1	3	0.9423	0.01500
CFBP	LM	Tansig-Tansig-Tansig	3-20-5-1	20	0.9600	0.00420
	BR	Purelin-Purelin-Purelin	3-8-5-1	5	0.9615	0.00335

## Data Availability

The data are available from the corresponding author upon request.
